# Generative Artificial Intelligence vs. Transformer and Benchmarking Against Deep/Machine Learning: Classification and Scientific Validation of Heart Failure Patients Using Women’s Transcriptomic Gene Data

**DOI:** 10.3390/diagnostics16071052

**Published:** 2026-04-01

**Authors:** Ekta Tiwari, Dipti Shrimankar, Krish Chaudhary, Luca Saba, Jasjit S. Suri

**Affiliations:** 1 Department of Computer Science and Engineering, Visvesvaraya National Institute of Technology, Nagpur 440010, India; ekta.tiwari.ai@gmail.com (E.T.); dshrimankar@cse.vnit.ac.in (D.S.); 2 Stroke Monitoring and Diagnostic Division, AtheroPoint LLC, Roseville, CA 95661, USA; chaudharykrish322@gmail.com; 3 Department of Computer Science and Engineering, Sikkim Manipal University, Majhitar, Rangpo 737136, India; 4 Department of Radiology, University of Cagliari, 09124 Cagliari, Italy; lucasabamd@gmail.com; 5 Department of Electrical and Computer Engineering, Idaho State University, Pocatello, ID 83209, USA; 6 Department of Innovation, Global Biomedical Technologies, Inc., Roseville, CA 95661, USA; 7 Symbiosis Institute of Technology, Nagpur Campus, Symbiosis International (Deemed University), Pune 412115, India

**Keywords:** heart failure, gene expression, differential expression analysis, hybrid deep learning, generalization, performance

## Abstract

**Backgrounds**: Accurate early classification of heart failure (HF) in women is challenging due to sex-specific gene expression and disease patterns, which traditional models overlook. We propose a generative artificial intelligence (GenAI)-based model for the classification of HF patients using acute myocardial infarction (AMI) gene expression data. **Objectives**: This study aims to design and scientifically validate a novel GenAI framework, benchmarking it against transformer, deep learning (DL), and machine learning (ML) architectures for robust HF classification using women’s transcriptomic data. **Methods**: Total 26 models designed: A novel wBio-GenAI model, two transformers (Xmers): token diffusion gene (wTDG-Xmer) and neurotopology (wNT-Xmer), 19 deep learning models which include convolutional neural network (CNN)-, long short-term memory (LSTM)- and extended LSTM (xLSTM)-based models, and four ML. The models applied differential expression analysis (DEA) which identifies differentially expressed genes (DEGs) from the public women’s microarray GSE57345 samples. Quality control was conducted. The GenAI system was scientifically validated, benchmarked, and statistically tested for reliability. **Results:** The wBio-GenAI achieved an accuracy of 98.21% and an area-under-the-curve (AUC) of 0.99. The wBio-GenAI is better than the mean of two Xmers by 4.67%, the mean of 19 DLs by 5.16%, and the mean of four MLs by 15.07%. The proposed model meets the regulatory requirements of having a difference < 10% between seen and unseen paradigms. **Conclusions:** The wBio-GenAI architecture captures the complex transcriptomic patterns, improving HF classification in women and advancing women-specific precision cardiovascular care.

## 1. Introduction

Heart disease is still the top cause of death worldwide and brings a financial toll to families and governments. One major type of heart disease is heart failure (HF), where the heart cannot pump enough blood to the body. It is very important to identify people at risk of HF early so they can get proper treatment and avoid worse outcomes [1]. But, heart disease is complicated because many patients also have common comorbidities like diabetes [2], high blood pressure [3], obesity, and renal [4] and erectile dysfunction [5]. These conditions often happen together and affect heart disease, thereby making it harder for doctors to assess risk using simple methods [6]. In women, unique factors like smaller heart size, hormonal changes, pregnancy history, and autoimmune diseases further affect risk. Current models struggle with these complexities, highlighting the need for more personalized and advanced approaches.

Traditional models often miss sex-specific molecular patterns, while the transformers (Xmers) and deep learning (DL) models including long short-term memory (LSTM) better handle sequences but still struggle with wide range of transcriptomic gene data. To directly address the limitations of existing cardiovascular disease (CVD) risk models [7], we designed and benchmarked the novel wBio-GenAI model against Xmers and hybrid deep learning (HDL) architectures that unifies unidirectional (uni) LSTM, bidirectional LSTM, and extended LSTM (xLSTM) variants with gating mechanisms, namely, cross gating (xLSTMcg) and exponential gating (xLSTMeg). To our knowledge, wBio-GenAI represents an early attempt to integrate generative energy-based modeling and dynamical systems for molecular-level, sex-specific heart failure prediction.

**Figure 1:**
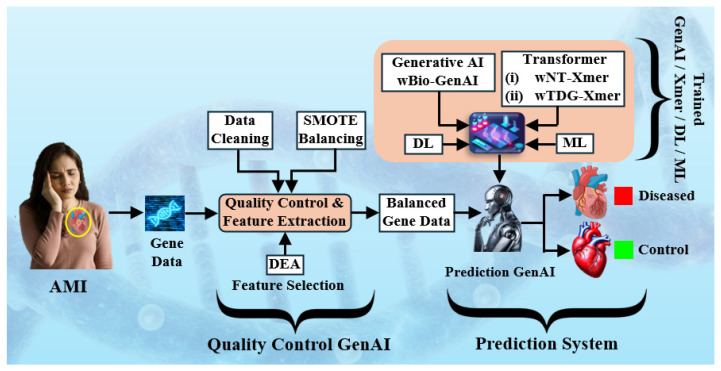
The online system for classification of womens heart failure patients. SMOTE: synthetic minority oversampling technique; DEA: differential expression analysis; ML: machine learning; DL: deep learning; Xmer: transformer; GenAI: generative artificial intelligence.

This innovation blends generative learning with an energy-based decision process, allowing the model to learn the structure of the data before making a classification judgment. Figure 1 illustrates the overall workflow of the proposed system for female HF classification using gene expression data. It highlights key steps including data cleaning, feature selection via differential expression analysis (DEA) [8], data balancing through synthetic minority oversampling technique (SMOTE) [9], and different types of classifier.

The GenAI has been a powerful tool in many domains such as natural language processing (NLP), computer vision, drug discovery, healthcare, finance, and engineering. This motivates us to adapt the GenAI framework for women’s CVD risk stratification. Therefore, we hypothesize that the wBio-GenAI is a powerful paradigm for classification of HF patients and it is the first time it is used in this domain. Our hypothesis includes that the wBio-GenAI is a better performer than the Xmers, DL, and ML models in both seen and unseen paradigms. DEA identified differentially expressed genes (DEGs) from the public women’s microarray GSE57345 samples. Quality control was conducted; the system was scientifically validated and benchmarked [10]. Further, statistical tests were conducted across the models for reliability. By observing the performance of all the models, we achieved the best model, i.e., wBio-GenAI, outperforming all the models. The benchmarking was done against Xmers, DL, and ML which confirms the superiority of our GenAI-based model.

This paper continues with Section 2 where the background literature is reviewed on cardiovascular risk factors in women and existing studies and prediction models. Section 3 presents the methodology. Section 4 presents the overall results, whereas Section 5 is the discussion which highlights the claims, benchmarking with clinical relevance and limitations, and Section 6 concludes key findings and future research directions. Additionally, Appendix A provides the equations for all performance metrics, while Appendix B presents a comprehensive list of all acronyms used throughout the manuscript, along with their definitions.

## 2. Background Literature

Recent advances in ML have significantly improved the diagnosis and classification of cardiovascular diseases, particularly those involving complex gene expression patterns. Saeed et al. [11] demonstrated that support vector machines (SVMs), artificial neural networks (ANNs), and random forests (RFs) can effectively diagnose coronary artery disease, with ANNs and RFs outperforming SVMs in capturing nonlinear relationships. However, their work primarily focused on clinical and physiological features, without fully exploiting transcriptomic data, limiting its applicability for molecular-level diagnostics. Tian et al. [12] integrated RF and ANN into a joint diagnostic model for heart failure, achieving improved accuracy. Nevertheless, their model was trained on relatively small datasets and lacked systematic validation across independent cohorts, raising concerns about its generalizability.

In acute myocardial infarction (AMI) studies, Yiffan et al. [13] developed an RF-based diagnostic model using ferroptosis-related genes from circulating endothelial cells, offering valuable biomarker insights but restricting analysis to a narrow set of genes, potentially overlooking broader molecular patterns. Xie et al. [14] identified metabolism-related genes associated with AMI, yet their approach relied on conventional statistical screening, which may not capture complex nonlinear dependencies among genes. Huang et al. [15] proposed a ferroptosis-based classification using ML, introducing a novel molecular framework, but their study did not compare multiple deep learning architectures or evaluate robustness under varying data availability.

Early-stage risk gene identification has also been explored. Fang et al. [16] constructed an SVM-based early diagnosis model for AMI, while Yang et al. [17] applied recursive feature elimination with SVMs to identify risk genes. Although both achieved promising results, they were limited to single-model ML approaches, lacked advanced data augmentation strategies, and did not explore DL techniques capable of learning hierarchical feature representations from high-dimensional gene expression data.

## 3. Methodology

In this study, we implemented a comprehensive methodological framework to develop and assess advanced DL models for gender-specific HF classification using transcriptomic data. Here, Section 3.1 describes the data and quality control, followed by Section 3.2 which describes the model architectures. Section 3.3 presents the experimental protocols which include the proposed hypothesis and the scientific validation. Section 3.4 shows the loss function and the hyper tuning parameters. Finally, Section 3.5 summarizes the performance metrics.

### 3.1. Data, Feature Extraction, and Quality Control

#### 3.1.1. Data

We have utilized the publicly available microarray dataset related to acute myocardial infarction (AMI). We use the dataset GSE57345 as D1:AMI345 (source: Perelman School of Medicine, Philadelphia, PA, USA, GSE57345). This dataset contains 177 diseased patients and 136 healthy control patients. The total number of genes (features) is 33,297. On the other hand, we have GSE66360 as D2:AMI360 (source: The Scripps Research Institute, La Jola, CA, USA, GSE66360). This dataset contains 49 diseased patients and 50 healthy control patients. The total number of genes is 54,675.

It is important to note that while these repositories provide high-quality gene expression profiles with basic clinical annotations, the available metadata are limited. The datasets include sex labels (male/female), which allowed us to perform sex-stratified analysis by selecting female patient samples. However, detailed female-specific clinical variables—such as hormonal status, menopausal status, pregnancy history, autoimmune conditions, and other sex-specific cardiovascular risk factors—are not available in the dataset metadata. Consequently, the modeling framework focuses on learning gene expression patterns derived from female patient samples rather than explicitly incorporating broader female biological variables. This limitation is acknowledged in the manuscript, and future work will aim to integrate transcriptomic data with richer clinical metadata when such datasets become available.

#### 3.1.2. Feature Extraction Using Differential Expression Analysis

**Figure 2:**
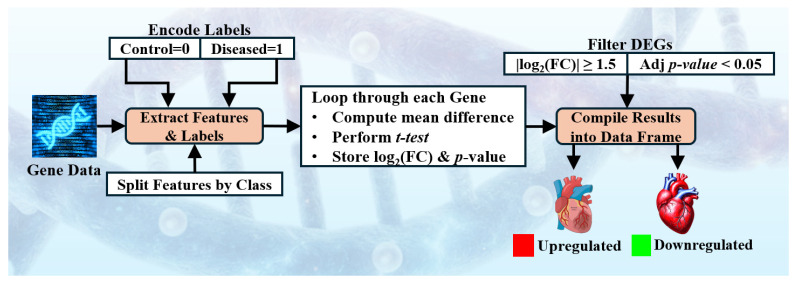
Block diagram of differential expression analysis. FC: fold change; Adj *p*-value: adjusted *p*-value.

We extracted samples exclusively from female patients to focus on sex-specific transcriptomic patterns. As shown in Figure 2, DEA was performed to identify DEGs, thereby reducing data dimensionality and emphasizing biologically relevant features.

**Figure 3:**
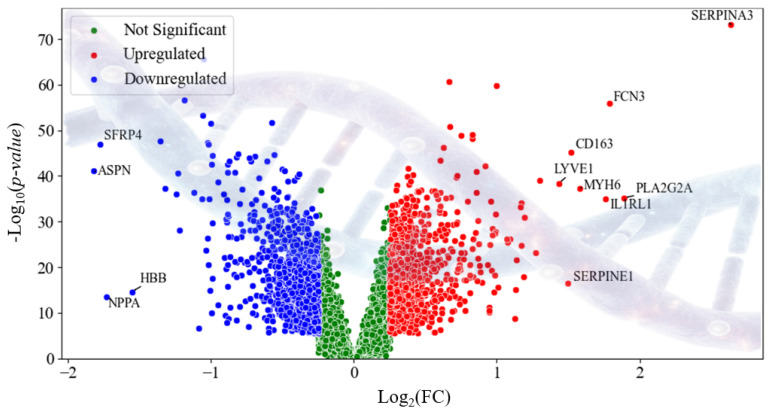
Volcano chart representing the high-risk genes obtained using DEA.

Using a custom Python-based (version 3.14) pipeline with SciPy and Statsmodels, log_2_ fold change and a two-sample *t*-test were computed for each gene. *p*-values were adjusted via the Benjamini–Hochberg procedure (False Discovery Rate) [18,19], and genes with |log_2_FC| ≥ 1.5 and adjusted *p*-value < 0.05 were selected and ranked by |log_2_FC| for model training. These filtered genes were then ranked by |log_2_FC| and used for model training. DEA results were validated against GEO2R, showing 100% consistency [20,21]. After the DEA, we observed the obtained high risk genes using volcano chart as shown in Figure 3. The detailed DEA architecture details with algorithm and sensitivity analysis is included in the Supplementary Material.

#### 3.1.3. Quality Control

To address the inherent class imbalance in the dataset, the SMOTE was applied [9,22]. For preprocessing, categorical class labels were encoded using a label encoder, and gene expression values were normalized to a common scale using the MinMaxScaler, standardizing the feature range across all samples and facilitating efficient model convergence [23,24]. To address the data leakage, all the above steps were strictly conducted in each training fold and not touching the testing fold, maintaining strict separation and unbiased performance assessment.

### 3.2. Model Architectures

In this section we describe the model architectures of the proposed wBio-GenAI model, Xmer models, and the DL models. We focus on wBio-GenAI architecture, its operation, and the benefits of biologically informed generative learning. The Xmer and DL models are included for comparison.

#### 3.2.1. wBio-GenAI

**Figure 4:**
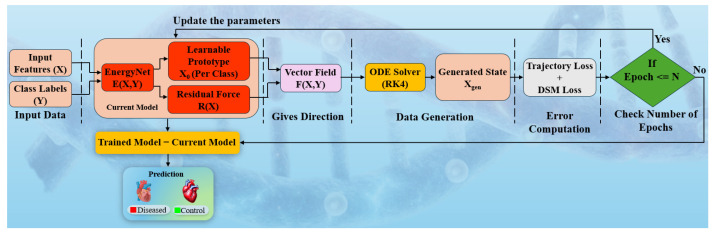
Architecture of wBio-GenAI model. DSM: denoising score matching, ODE: ordinary differential equation, RK4: fourth-order Runge–Kutta.

The proposed wBio-GenAI framework is designed to perform robust disease classification from high-dimensional gene expression data by jointly modeling class-conditioned energy landscapes and continuous-time dynamical trajectories. As shown in Figure 4, the input to the model is a gene expression feature vector $X\in R^{n_{\mathrm{genes}}}$, where $n_{\mathrm{genes}}$ denotes the number of genes, along with a corresponding class label Y∈{0,1}, representing Control and Diseased categories, respectively. Prior to training, all input features are scaled to the range [0, 1] using min–max normalization to ensure numerical stability.

At the core of the architecture lies EnergyNet, a class-conditioned energy-based network that learns a scalar energy function E(X,Y)∈R. The class label Y is first mapped to a low-dimensional latent representation using a learnable embedding layer, which enables the model to capture semantic relationships between classes. This embedded label is concatenated with the input feature vector X and passed through a multilayer perceptron (MLP) composed of fully connected layers with Exponential Linear Unit (ELU) activations. The resulting scalar energy quantifies the compatibility between an input sample and a specific class, with lower energy values indicating higher class consistency.

To support generative modeling and enforce biologically meaningful transitions, the framework introduces learnable class prototypes $X_{0}^{\left(Y\right)}\in R^{n_{\mathrm{genes}}}$, where each prototype represents an initial latent biological state for class Y. These prototypes are trainable parameters and serve as starting points for trajectory generation in feature space. By conditioning the dynamics on class-specific prototypes, the model ensures that generated trajectories remain anchored to biologically plausible class representations.

The evolution of samples in feature space is governed by a vector field F(X,Y), which defines the instantaneous direction of change for a given state X and class label Y. This vector field consists of two complementary components. The first is a conservative force, defined as the negative gradient of the energy function with respect to the input features, $-\nabla_{X}E(X,Y)$. This force encourages samples to move toward regions of lower energy, thereby aligning them with class-consistent manifolds. The second component is a residual force R(X), modeled using a learnable, normalized interaction matrix that captures gene–gene dependencies and non-conservative biological interactions. The final vector field is thus expressed as

(1) $$F(X,Y)=-\nabla_{X}E(X,Y)+R(X)$$

To model continuous biological dynamics, the defined vector field is integrated over time using a fourth-order Runge–Kutta (RK4) ordinary differential equation (ODE) solver. Starting from the class-specific prototype $X_{0}^{\left(Y\right)}$, the solver iteratively updates the state according to the vector field F(X,Y), producing a generated sample $X_{\mathrm{gen}}$. This continuous-time formulation allows the model to simulate smooth transitions in gene expression space, reflecting gradual biological progression rather than abrupt changes.

The training objective combines two complementary loss functions to jointly optimize the energy landscape and the dynamical system. The first is the denoising score matching (DSM) loss, which trains EnergyNet to approximate the score function by matching the gradient of the energy function on noisy inputs to the true denoising direction. The second is a trajectory reconstruction loss, which penalizes the mean squared error between the generated sample $X_{\mathrm{gen}}$ and the corresponding real input X. Together, these losses encourage both accurate local gradient estimation and global trajectory consistency, and the total loss is minimized using the Adam optimizer.

During inference, classification is performed using an energy-based scoring mechanism. For a given input sample X, the energy values are computed for both class labels, yielding E(X,0) and E(X,1). The final decision score is defined as the energy difference E(X,0)−E(X,1), where higher values indicate stronger evidence for the diseased class. A decision threshold is selected based on receiver operating characteristic (ROC) analysis on validation data, enabling robust classification while accounting for class imbalance and inter-cohort variability.

One of the most important things about wBio-GenAI model is its biological interpretability. Its architecture explicitly incorporates principles inspired by gene regulation. Adaptive feature gating allows the model to emphasize or suppress individual genes according to their relevance to a phenotype, reflecting the fact that not all genes contribute equally to a cellular state or disease. Class-specific prototypes anchor the trajectories of gene expression in biologically plausible regions, simulating gradual transitions that mirror cellular progression from healthy to diseased states. Additionally, the residual vector field captures gene–gene dependencies, modeling non-linear interactions observed in real biological networks. Together, these components enable the model to learn from high-dimensional data while maintaining alignment with underlying biological processes.

#### 3.2.2. wNT-Xmer

**Figure 5:**
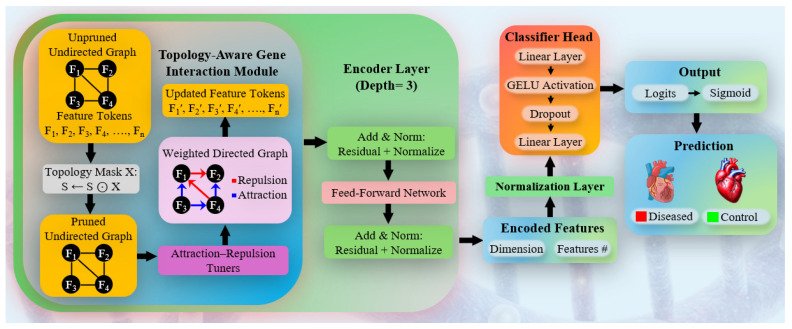
Architecture of neurotopology transformer (wNT-Xmer), illustrating the topology-aware interaction module, multi-layer encoder, and classification head combined with logist/sigmoid for discriminating diseased/control samples.

The wNT-Xmer first builds an undirected graph that shows how genes are related to each other using the input feature tokens. A topology mask is then applied to remove weak or biologically meaningless connections, resulting in a smaller and more meaningful gene network (Figure 5). This refined graph is processed by a topology-aware interaction module, where attraction and repulsion mechanisms learn whether gene relationships are supportive or antagonistic. These learned relationships are used to update the gene feature representations, which are then passed through a three-layer Xmer encoder. Each layer uses attention, residual connections, normalization, and feed-forward processing to progressively refine the features. The final representations are normalized and fed into a lightweight classifier with linear layers, GELU activation, and dropout, followed by a sigmoid output layer for binary disease versus control prediction.

#### 3.2.3. wTDG-Xmer

**Figure 6:**
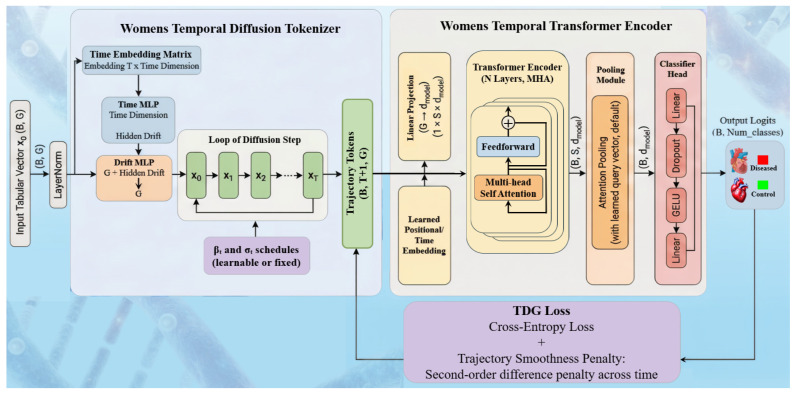
Architecture of temporal diffusion gene transformer (wTDG-Xmer); B: batch size, G: number of Genes, T: time steps, S: sequential length, d_model_: dimension of model, X0: input state, XT: final state, X1, X2: intermediate states, MHA: multi-head attention, β_t_: the coefficient controlling the drift strength at step t, σ_t_: the coefficient controlling the noise variance (stochasticity) at step t, TDG: temporal diffusion gene, MLP: multi-layer perceptron, GELU: Gaussian error linear unit.

As shown in Figure 6, the wTDG-Xmer model starts by converting each fixed gene-expression vector into a short sequence using a diffusion-based tokenizer. At each diffusion step, the tokenizer adds controlled noise along with time-related information and a small drift network, creating a sequence of representations that explore small variations around the original gene profile. This sequence is then passed to a temporal Xmer encoder, where the time steps are mapped into a common feature space, combined with positional information, and processed through multiple attention and feed-forward layers. After temporal encoding, the sequence is summarized using pooling methods such as mean pooling, last-step pooling, or attention-based pooling. The final pooled representation is normalized and fed into a lightweight classifier to predict disease versus control. An optional auxiliary loss can be added to ensure smooth transitions across diffusion steps and maintain temporal consistency.

#### 3.2.4. DL Models

**Figure 7:**
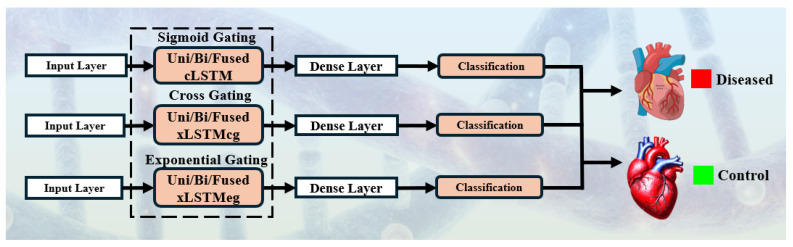
Model architectures with different gating mechanisms using (uni/bi/hybrid) for HF patient classification. Uni: unidirectional; Bi: bidirectional; cLSTM: conventional long short-term memory; xLSTMcg: extended LSTM cross gating; xLSTMeg: extended LSTM exponential gating.

For benchmarking we implemented a basic three-hidden-layer CNN model and 18 (HDL and SDL) models derived from three core recurrent cell types: classical LSTM with sigmoid gating (cLSTM), cross-gated extended LSTM (xLSTMcg), and exponential-gated extended LSTM (xLSTMeg), as illustrated in Figure 7. Each model began with an input layer receiving pre-processed gene expression vectors, followed by one or more recurrent layers configured in unidirectional or bidirectional, i.e., SDL, or fusion of (Uni-Bi/Bi-Uni), i.e, HDL to capture both forward and backward temporal dependencies. The recurrent blocks employed either single-layer (SDL) or stacked two-layer (HDL) setups, with the first recurrent stage typically using 128 units and, in deeper variants, a second stage of 64 units. Dropout layers were applied after recurrent blocks to mitigate overfitting. The outputs of the recurrent layers were passed to a fully connected dense layer, culminating in a single sigmoid neuron for binary classification of patients into diseased or control groups. The detailed architecture diagram is included in the Supplementary Material Figure S1.

The xLSTMcg architecture introduced cross-cell gating to enhance inter-dimensional feature communication, whereas xLSTMeg applied exponential gating to dynamically regulate information retention, making it more adaptable to heterogeneous transcriptomic sequences. This unified architecture design allowed systematic comparison between conventional gating, cross gating, and exponential gating mechanisms under consistent experimental settings.

The cLSTM architecture regulates information flow through three sigmoid-controlled gates at each timestep: the input gate, which determines how much new information from the current input and the previous hidden state is incorporated into the cell state; the forget gate, which modulates the retention of prior cell state information; and the output gate, which controls the influence of the updated cell state on the hidden output. A candidate cell state is computed using hyperbolic tangent activation, and the final cell and hidden states are obtained through gated combinations of preserved and newly computed values, enabling selective information retention and mitigating gradient decay in long sequences.

The xLSTMcg extends this mechanism by partitioning the hidden state into multiple parallel substreams. Each substream processes the current input, a concatenated representation of all substream outputs from the previous timestep, and a cross-influence term capturing learned inter-stream dependencies. These inputs are passed through sigmoid-activated gates that regulate information flow and promote contextual sharing across substreams. The resulting substream-specific hidden states are concatenated to form the overall hidden representation, thereby enhancing the model’s capacity to capture diverse and interdependent temporal patterns.

The xLSTMeg further modifies the gating mechanism by replacing the sigmoid activation of the input gate with an exponential function to amplify the response to significant input features, while the forget and output gates retain their standard sigmoid activations. The cell state update and hidden state computation follow the conventional LSTM formulation, with the amplified input gating improving the model’s ability to capture and represent salient variations in sequential data. 

### 3.3. Experimental Protocols

Our experiments were designed to analyze the performances and the novelty of the wBio-GenAI framework over conventional modeling approaches.

#### 3.3.1. Experimental #1: Cross-Validation for Seen Evaluation

In this protocol we train the models using the seen data, i.e., training and testing on D1 and D2. We train the models using K5 protocol. This shows how well the model learns when there is no distributional shift. The result of this experiment is presented in Section 4.

#### 3.3.2. Experimental #2: Unseen Evaluation for Scientific Validation

In this protocol we train the models using D1 dataset and test on D2 dataset. This shows how our models perform in external data. This shows the generalization and robustness of our models. The drop in accuracy between the seen and unseen settings should be <10% as per the regulatory requirements. The result of this experiment is presented in Section 4.2.1.

#### 3.3.3. Experiment #3: K-Effect for Scientific Validation

We evaluated model stability and generalization using multiple k-fold cross-validation schemes with k values of 2, 3, 4, 5, and 10. For each k, the dataset was split into k equal folds; in each iteration, the model was trained on k − 1 folds and validated on the remaining one-fold. This process was repeated until each fold had served once as the validation set. Additionally, we performed a total training and testing (TT) run using the entire dataset without fold-based splitting. Averaging performance across folds provided robust estimates and minimized bias from data partitioning. The detailed comparative results for this experiment are reported in Section 4.2.2.

#### 3.3.4. Experiment #4: Memorization vs. Generalization Effect for Scientific Validation

To assess how training data quantity affects model outcomes, we trained models on varying proportions of the dataset, ranging from 10% to 100% including the total training and testing (TT) condition. Performance metrics including accuracy and AUC generally improved as more data was used for training, demonstrating the positive correlation between training set size and model generalization capability. This finding underscores the importance of sufficient training samples for developing reliable predictive models in transcriptomic analysis. The detailed comparative results for this experiment are reported in Section 4.2.3.

### 3.4. Loss Function and Hyperparameter Tuning

To train our models for binary classification of HF samples, we employed the Binary Cross-Entropy (BCE) loss function [25]. This loss is well-suited for two-class problems where the model outputs a probability score indicating the likelihood of each sample belonging to the positive class [26]. The BCE loss measures the dissimilarity between the accurate actual label and the predicted probability and is defined as:

(2) $$L=-\frac{1}{N}\sum_{i=1}^{N}\left[y_{i}\log\left({\hat{y}}_{i}\right)+\left(1-y_{i}\right)\log\left(1-{\hat{y}}_{i}\right)\right]$$

where N is the number of samples, $y_{i}\in (0,\;1)$ is the true label for sample i, ${\hat{y}}_{i}\in \left(0,\;1\right)$ is the predicted probability for the positive class. This loss encourages the model to output probabilities close to 1 for positive samples and close to 0 for negative samples. During training, the loss is minimized using the Adam optimizer, with early stopping applied to prevent overfitting. In cases of mild class imbalance, the BCE loss still performed robustly, especially when combined with techniques like SMOTE during data preprocessing. The simplicity and mathematical properties of BCE make it a reliable choice for deep neural networks in biomedical classification tasks [27].

### 3.5. Hyperparameter Tuning

To improve model performance, we carried out hyperparameter tuning using grid search along with early stopping to find the best combination of parameters like learning rate, number of neurons, batch size, and dropout rate. This process helped the models learn more efficiently and reduced overfitting.

### 3.6. Performance Metrics

To evaluate our classification models, we used key performance metrics including accuracy, precision, recall (true positive rate), specificity (true negative rate), false positive rate, false negative rate, and F1-score. Accuracy measures the overall correctness, while precision indicates the reliability of positive predictions. Recall (or sensitivity) reflects the model’s ability to identify true positives, and specificity measures how well it recognizes true negatives. False positive and false negative rates capture error proportions. The F1-score balances precision and recall providing a single performance measure. Together, these metrics provide a comprehensive understanding of the model’s effectiveness, especially in sensitive biomedical contexts where correctly identifying positive cases is crucial. The formulas and detailed explanations for these metrics are provided in Appendix A.

## 4. Results

**Figure 8:**
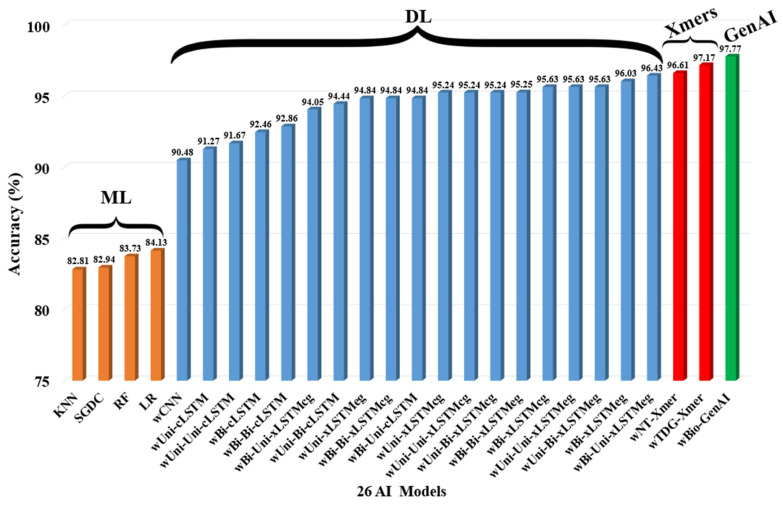
Accuracy performance of all the models in seen paradigm (training and testing on D1). ML: machine learning; CNN: convolutional neural network; KNN: k-nearest neighbors; SGDC: stochastic gradient descent classifier; RF: random forest; LR: logistic regression; Uni: unidirectional; Bi: bidirectional; cLSTM: conventional long short-term memory; cg: cross gating; eg: exponential gating; NT: neurotopology; wTDG: token diffusion; Xmers: transformers; GenAI: generative artificial intelligence.

The performance of all models was evaluated comprehensively, highlighting classification accuracy, precision, recall, and other relevant metrics across the models. To identify the best-performing model, we compared the classification performance in seen paradigm, i.e., training and testing on D1 using K5 protocol of various architectures.. These architectures include wBio-GenAI, Xmers, traditional ML models, and DL models. Figure 8 illustrates the results showing accuracy across all the models. The visualizations clearly demonstrate that the wBio-GenAI model outperforms all the models, achieving the highest accuracy of 97.77% and an AUC of 0.99. These results highlight the superior capability of the wBio-GenAI architecture in accurately classifying HF using transcriptomic gene data.

### 4.1. Performance Evaluation and Reliability Analysis

#### 4.1.1. ROC Analysis

**Figure 9:**
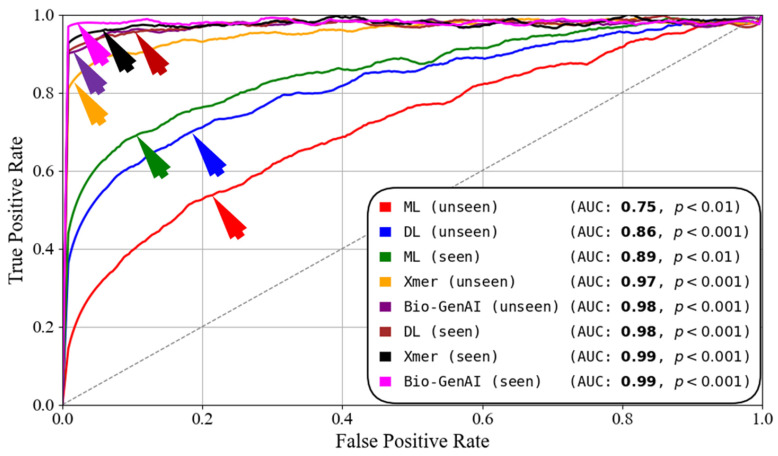
ROC curves comparing seen and unseen evaluations. Seen: training and testing on D1; unseen: training on D1 and testing on D2.

Figure 9 shows the ROC curve comparison of the wBio-GenAI, Xmer, DL and ML. We can see the wBio-GenAI model achieved an AUC of 0.99 and 0.98 in seen and unseen paradigms, respectively. The Xmers also have performed well by achieving an AUC of 0.99 and 0.97 in seen and unseen paradigms, respectively. This clearly shows that the wBio-GenAI model outperforms the Xmers in unseen and seen settings. Similarly, the DL has achieved an AUC of 0.98 and 0.86 in seen and unseen paradigms, respectively. The ML has achieved an AUC of 0.89 and 0.75 for seen and unseen paradigms respectively. Although the DL and ML have performed well in seen paradigm, in unseen paradigm they have a significant difference. This shows that the wBio-GenAI model is superior to Xmers, DL, and ML models. This shows the advantage of generative and energy-based learning in capturing complex gene expression patterns and achieving superior cardiovascular disease discrimination.

#### 4.1.2. Reliability Analysis Using Statistical Tests

In the context of transcriptomic gene data analysis, assessing the reliability and robustness of classification models is crucial [28,29]. Raw performance metrics such as accuracy, precision, or F1-score provide an overall measure of effectiveness, but they do not fully capture the statistical significance or consistency of the observed performance across datasets or experimental replicates. The reliability tests show, except the ML, that almost all the models, especially the GenAI and Xmer architectures, produce highly stable results across repeated runs. The *p*-values are reported in Table 1. These values indicate that performance differences are systematic rather than random, confirming strong reproducibility.

### 4.2. Scientific Validation

To ensure the scientific validity and robustness of our model, we performed three sets of unique experiments: (i) the unseen analysis (Section 4.2.1), (ii) K-effect (Section 4.2.2 ), and (iii) memorization vs. generalization effect (Section 4.2.3).

#### 4.2.1. Unseen Analysis for Scientific Validation

As shown in Table 2, the unseen paradigm consists of the combination of training on D1 and testing on D2 (Tr(D1)–Te(D2)), which evaluates cross-dataset generalization. The performance degradation is observed across all models during unseen analysis, as expected. Despite this, wBio-GenAI maintains the highest unseen accuracy (95%) and AUC (0.98), demonstrating superior robustness to distribution shifts. Also, the performance drop on unseen data is 2.77%, because wBio-GenAI does not rely solely on surface-level patterns in the data. Instead, it learns smooth, biologically guided energy landscapes and continuous gene-expression trajectories, which act as strong regularizers. This allows the model to stay stable even when the data distribution changes, leading to much better generalization than purely discriminative models. As compared to wBio-GenAI, transformer-based models, including wNT-Xmer and wTDG-Xmer, show performance drops of 4.61% and 6.17% respectively, reflecting their reliance on discriminative learning and sensitivity to dataset-specific patterns. While comparing them to DL and ML models, their performance drop ranges between 10% and 20% which violates the regulatory requirements.

The performance drop (PD) further highlights model stability, where wBio-GenAI exhibits the lowest degradation (2.77%), confirming its strong generalization capability. Overall, the results validate that the proposed wBio-GenAI framework consistently outperforms Xmer-based and variational generative models, particularly in unseen, cross-cohort evaluation scenarios critical for real-world clinical deployment.

Current regulatory guidance does not prescribe a fixed numerical threshold for performance differences between seen and unseen data; instead, it emphasizes independent validation, strict train–test separation, and transparent performance reporting to demonstrate robustness and generalization (U.S. Food and Drug Administration Good Machine Learning Practice (FDA GMLP) (https://www.fda.gov/medical-devices/software-medical-device-samd/good-machine-learning-practice-medical-device-development-guiding-principles?, accessed on 20 January 2026); G7 Principles for the Evaluation of Artificial Intelligence and Machine Learning–Enabled Medical Devices (G7 AI/ML Evaluation Principles)). In alignment with these expectations, our evaluation framework enforces independent testing and avoids optimization on unseen datasets. Accordingly, the reported results represent pre-regulatory evidence of generalization, rather than formal regulatory compliance or approval.

#### 4.2.2. K-Fold Cross-Validation for Scientific Validation

**Figure 10:**
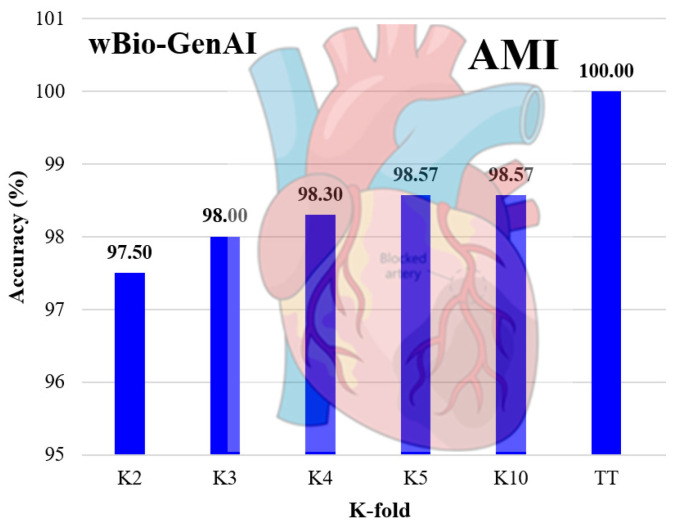
K-effect of wBio-GenAI model using AMI gene data.

As shown in Figure 10, we can see the accuracy gradually improves as K increases. There is a slight performance drop with the lower folds K2, K3. With higher folds K5, K10 we can see there is more stable accuracy. With higher folds, the model is trained on a larger and more diverse portion of the data, allowing the energy landscape and biological trajectories to be learned more accurately and stably. Under ideal conditions of full data sets used for training and testing (called as TT protocol), we see that wBio-GenAI shows almost 100% accuracy. This shows complete alignment between the learned energy model and the available data, validating the model’s scientifically consistent learning behavior rather than overfitting.We have observed similar behavior for Xmers and the DL models.

#### 4.2.3. Memorization vs. Generalization for Scientific Validation

**Figure 11:**
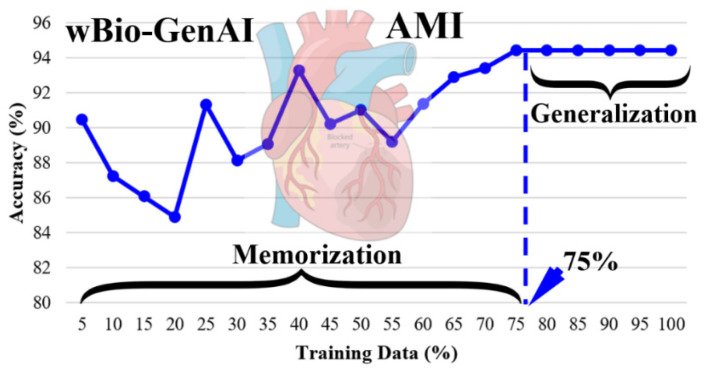
Memorization vs. generalization effect of wBio-GenAI model using AMI gene data.

One of the most important criteria for scientific validation of the AI model is evaluating the memorization vs. generalization experiment, which informs us about the ability of the model to generalize with a specific amount of training data sets. As shown in Figure 11, the wBio-GenAI model requires 75% of the data for generalization. This is because it does not learn individual samples directly. Instead, it learns shared class-level energy landscapes and smooth biological trajectories, which capture the underlying structure of gene expression rather than dataset-specific noise. Once these class-consistent patterns are learned, additional data mainly repeats similar biological information, leading to stable performance and early generalization. This indicates the generalization phase. Beyond these thresholds, the accuracy plateaued. We can see that the wBio-GenAI shows strong generalization once sufficient training data is provided. Similarly, we have observed the generalization behavior requiring training data in between 50% and 75% for the Xmers and the DL models.

## 5. Discussion

The aim of this study is to design a classification system embedded with generative AI and transformer-based models, and benchmark against deep learning and machine learning models. We scientifically validated these systems. We therefore present in this section: the main claims of this study, comparison of our design against the previous studies of this type, followed by strengths, weaknesses, and future extensions.

### 5.1. Claims

The experimental framework incorporates the following key components: (i) SMOTE was applied strictly within each training fold to address class imbalance while preventing information leakage; (ii) DEA was performed on a fold-wise basis to identify biologically meaningful genes using only training data (Supplementary Material Figures S2 and S3); (iii) the seen paradigm evaluated model performance on data originating from the same source to assess learning under controlled conditions; (iv) the unseen paradigm tested cross-dataset generalization using independent cohorts from different institutions and populations; (v) the K-effect experiment analyzed model stability and performance sensitivity across varying K-fold cross-validation settings; (vi) the generalization effect experiment quantified the model’s ability to distinguish memorization from true biological generalization under seen–unseen conditions; (vii) Reliability and stability analysis assessed consistency across folds, datasets, and repeated runs to ensure robust and reproducible performance; (viii) demonstrated the ability of wBio-GenAI outperforming all the models; and (ix) all experiments were executed on a high-end GPU cluster, enabling efficient training of generative models, transformer models, and legacy systems, and ensuring computational scalability.

### 5.2. Benchmarking

We are trying to determine the effectiveness of the wBio-GenAI framework. That is why we conducted a study of other existing methodologies for classifying gene expression. This is shown in Table 3. It is not only about accuracy; it also analyses other dimensions like feature selection, model design, result validation, model robustness, and other dimensions of performance. Most of the other research makes use of feature selection techniques, for example, differential expression analysis, transcriptome-wide association study (TWAS), recursive feature elimination, or network-based methods like weighted gene co-expression network analysis (WGCNA), coupled with some classifiers like logistic regression, random forests, support vector machine, or shallow neural network. They seem to perform comparatively better when the data is sampled from the same distribution as the data they had previously been trained on. However, a lot of this work does not perform more rigorous evaluation. They rarely get evaluated on a holdout, or unseen data. Generalization is not well studied, the robustness of a system with noise is rarely evaluated, and the soft or a method’s scaling to use multiple GPUs is not studied at all.

In contrast, the proposed framework takes a broader approach. As shown in Figure 1, it combines DEA with a GenAI architecture enhanced attention, which helps the model capture richer feature interactions while remaining stable across folds. More importantly, the evaluation protocol explicitly includes unseen testing, generalization effect, K-effect experiments, and reliability checks. The gains are visible in the numbers. Unseen accuracy reaches 94.64% with an AUC of 0.99, while performance on seen data remains strong at 98.21% accuracy and a 0.99 AUC. Taken together, the benchmarking suggests that wBio-GenAI is not just another incremental model, but a more complete and resilient option for gene-based cardiovascular disease classification.

### 5.3. A Special Note on Generative Artificial Intelligence

Most DL classifiers are trained to draw a clean line between classes and stop there. The wBio-GenAI model takes a slower, and perhaps more cautious, route. Instead of jumping straight to a decision boundary, it learns what the data itself looks like by using denoising score matching. Noisy gene expression profiles are gradually nudged toward a more realistic biological shape, almost the way a lab technician might mentally correct a messy readout after seeing enough samples. What emerges is not just a classifier, but a sense of the underlying structure beyond surface-level correlations that can break when the dataset changes.

That generative perspective turns out to be useful when decisions are made using energy differences rather than raw prediction scores [31]. The model seems less rattled by shifts across cohorts or experimental settings, which is a common headache in transcriptomic studies. Of course, generative learning is not a cure-all, but in biomedical contexts marked by small sample sizes and noisy measurements, it offers something different. Moreover, the framework fits naturally within a broader view of generative AI, one that values distribution learning, noise correction, and data restoration instead of focusing only on optimizing a discriminative objective.

Building on this generative perspective, wBio-GenAI also offers biological interpretability. The model’s adaptive feature gating highlights the genes that contribute most to classification, while the trajectories generated from class-specific prototypes reveal coordinated patterns of gene expression associated with disease progression. To provide concrete validation, differential expression analysis across HCM and AMI datasets identified key genes in the range of 90 to 100 for each fold. Some key genes are: SERPINA3, PLA2G2A, ASPN, FCN3, SFRP4, IL1RL1, NPPA, MYH6, HBB, CD163, SERPINE1, LYVE1, FRZB, EIF1AY, OGN, VSIG4, and others. Pathway-level analyses using gene ontology (GO) [32,33] (Supplementary Material Figure S4) and Kyoto Encyclopedia of Genes and Genomes (KEGG) [34,35] (Supplementary Material Figure S5) further showed that these genes converge on biologically meaningful processes and pathways, including extracellular matrix organization, neutrophil activation, complement activation, vascular wound healing, complement and coagulation cascades, the renin–angiotensin system, oxidative stress responses, and hematopoietic cell lineage. These results indicate that the model’s learned features and generative trajectories reflect real biological mechanisms, linking the mathematical constructs of energy landscapes and score matching to interpretable disease-relevant patterns. Furthermore, details on the genes and the pathway-level analyses is available in the Supplementary Material.

### 5.4. Strengths, Weakness, and Extensions

The framework’s main strength lies in its ability to generalize across datasets drawn from different institutions and populations, a setting where many gene-based models tend to struggle. Careful separation of training and testing data, even during preprocessing, helps prevent information leakage and keeps performance estimates honest. The energy-based decision rule offers a stable and transparent criterion, while denoising trajectory learning improves robustness to noise and batch effects that are common in transcriptomic data. Moreover, the same architecture can be applied to other disease contexts without structural changes, which makes it practical beyond a single use case.

There are, however, some clear limitations. Generative training is computationally more demanding than conventional discriminative approaches, particularly during trajectory learning. Relying on pre-selected differentially expressed genes may also constrain the discovery of less obvious biomarkers. Finally, although energy-based decisions enhance robustness, they can be harder to interpret than feature-attribution methods used in simpler machine learning models, which may limit their appeal in clinically oriented settings.

Future extensions of this work include integrating multi-omics data, such as proteomics and metabolomics, to further enhance disease characterization. Incorporating attention mechanisms within the score network may improve feature-level interpretability [36,37]. Additionally, the framework can be adapted for semi-supervised or self-supervised learning to exploit unlabeled data [38,39,40,41]. Finally, translating the model into a clinical decision-support system with real-time inference and uncertainty estimation represents a promising direction for practical deployment.

## 6. Conclusions

In this work, we addressed the problem of robust women’s cardiovascular disease classification using high-dimensional gene expression data. We proposed a GenAI model, namely wBio-GenAI, which follows an energy-based learning paradigm. In this study: (i) we designed a rigorous experimental pipeline that combines fold-wise differential expression analysis, strict data isolation, and SMOTE-based imbalance handling to ensure scientifically valid evaluation; (ii) we proposed an energy-based generative architecture trained via denoising score matching, enabling the model to learn intrinsic transcriptomic structure rather than relying solely on discriminative decision boundaries; and (iii) we conducted extensive validation under seen, unseen, K-effect, and memorization versus generalization. The experimental results show that the wBio-GenAI outperforms the transformers, DL and ML models in seen and cross-dataset/unseen scenarios. The wBio-GenAI shows strong stability, reliability, and robust performance in unseen settings. Therefore, all the findings highlight the importance of generative AI for the classification of heart disease patients.

## Figures and Tables

**Table 1:** Statistical tests over selected models.

SN	AI Models	Paired *t*-Test	Wilcoxon	Mann Whitney
1	RF	*p* < 0.0001	*p* < 0.0001	*p* < 0.04
2	SGDC	*p* < 0.002	*p* < 0.002	*p* < 0.04
3	KNN	*p* < 0.5	*p* < 0.5	*p* < 0.7
4	LR	*p* < 0.7	*p* < 0.7	*p* < 0.8
5	wCNN	*p* < 0.003	*p* < 0.0001	*p* < 0.04
6	wBi-xLSTMeg	*p* < 0.0005	*p* < 0.0005	*p* < 0.003
7	wBi-Uni-xLSTMeg	*p* < 0.0005	*p* < 0.0005	*p* < 0.002
8	wBi-Uni-xLSTMcg	*p <* 0.0004	*p* < 0.0005	*p <* 0.003
9	wBi-Uni-cLSTM	*p* < 0.0004	*p* < 0.0003	*p* < 0.004
10	wUni-Bi-xLSTMeg	*p* < 0.0005	*p* < 0.0004	*p* < 0.0005
11	wUni-Bi-xLSTMcg	*p* < 0.0004	*p* < 0.0004	*p* < 0.0005
12	wUni-Bi-cLSTM	*p* < 0.0005	*p* < 0.0005	*p* < 0.0004
13	wTDG-Xmer	*p* < 0.0001	*p* < 0.0001	*p* < 0.0001
14	wNT-Xmer	*p* < 0.0001	*p* < 0.0001	*p* < 0.0001
15	wBio-GenAI	*p* < 0.0001	*p* < 0.0001	*p* < 0.0001

RF: random forest; wCNN: convolutional neural network; KNN: k-nearest neighbor; LR: logistic regression; SGDC: stochastic gradient descent classifier; Bi: bidirectional; Uni: unidirectional; xLSTM: extended long-short memory; eg: exponential gating; cg: cross gating; wTDG: token diffusion gene transformer; wNT-Xmer: neurotopology transformer.

**Table 2:** Seen Vs. unseen Results of wBio-GenAI, Xmers, DL, and ML models.

SN		Models	Seen: Tr(D1)-Te(D1)					Unseen: Tr(D1)-Te(D2)					PD ACC (%)
			ACCU (%)	PREC (%)	REC (%)	FS (%)	AUC [0–1]	ACCU (%)	PREC (%)	REC (%)	FS (%)	AUC [0–1]	
	1	wBio-GenAI	97.77	98.88	96.59	97.77	0.99	95.00	94.12	96.00	95.05	0.98	2.77
Xmer	2	wNT-Xmer	96.61	93.61	100.00	96.70	0.98	92.00	87.50	98.00	92.45	0.98	4.61
	3	wTDG-Xmer	97.17	97.77	96.59	97.14	0.99	91.00	93.62	88.00	90.72	0.95	6.17
	Mean (μ _Xmer_)		96.89	95.69	98.30	96.92	0.99	92.67	91.75	94.00	92.74	0.97	4.22
	SD (σ _Xmer_)		±0.28	±2.08	±1.71	±0.22	±0.01	±1.70	±3.01	±4.32	±1.78	±0.01	±1.42
	4	wCNN	90.48	89.31	92.13	90.70	0.94	80.21	81.22	84.31	82.46	0.86	10.27
DL	5	wUni-cLSTM	91.27	91.34	91.34	91.34	0.96	81.03	83.90	77.17	80.39	0.88	10.24
	6	wBi-cLSTM	92.46	94.00	89.76	92.31	0.97	83.10	81.31	86.30	83.73	0.91	9.36
	7	wUni-xLSTMeg	94.84	94.53	95.28	94.90	0.98	81.43	94.07	67.40	78.53	0.86	13.41
	8	wUni-xLSTMcg	95.24	95.28	95.28	95.28	0.98	78.57	91.95	62.99	74.77	0.86	16.67
	9	wBi-xLSTMcg	95.63	96.77	94.49	95.62	0.99	75.16	70.43	87.40	78.00	0.82	20.47
	10	wBi-xLSTMeg	96.03	96.80	95.28	96.03	0.99	80.95	87.76	72.28	79.27	0.85	15.08
	11	wUni-Uni-cLSTM	91.67	92.06	91.34	91.70	0.97	77.94	88.72	64.41	74.64	0.86	13.73
	12	wBi-Bi-cLSTM	92.86	95.04	90.55	92.74	0.97	76.75	76.31	78.11	77.20	0.86	16.11
	13	wBi-Uni-xLSTMcg	94.05	95.16	92.91	94.02	0.98	81.90	80.60	84.41	82.46	0.86	12.15
	14	wUni-Bi-cLSTM	94.44	94.49	94.49	94.49	0.98	82.78	85.30	79.53	82.31	0.88	11.66
	15	wBi-Bi-xLSTMcg	94.84	93.18	96.85	94.98	0.98	75.87	79.50	70.24	74.58	0.82	18.97
	16	wBi-Uni-cLSTM	94.84	97.50	92.13	94.74	0.98	78.81	76.14	84.41	80.06	0.85	16.03
	17	wUni-Uni-wwxLSTMcg	95.24	96.00	94.49	95.24	0.99	78.73	90.87	64.25	75.28	0.86	16.51
	18	wUni-Bi-xLSTMcg	95.24	95.28	95.28	95.28	0.98	80.40	74.62	92.60	82.64	0.88	14.84
	19	wBi-Bi-xLSTMeg	95.25	92.59	98.43	95.42	0.99	77.94	81.71	72.44	76.79	0.86	17.31
	20	wUni-Uni-wxLSTMeg	95.63	95.31	96.06	95.69	0.99	76.11	78.40	72.60	75.39	0.79	19.52
	21	wUni-Bi-xLSTMeg	95.63	95.31	96.06	95.69	0.99	81.27	82.13	80.31	81.21	0.88	14.36
	22	wBi-Uni-xLSTMeg	96.43	96.83	96.06	96.44	0.99	76.27	85.44	63.78	73.04	0.85	20.16
	Mean (μ _DL)_		94.32	94.57	94.12	94.35	0.98	79.22	82.65	76.05	78.57	0.86	15.1
	SD (σ _DL_)		±1.64	±1.99	±2.23	±1.63	±0.01	±2.39	±6.06	±8.87	±3.27	±0.03	±0.75
ML	23	RF	83.73	80.28	89.76	84.76	0.92	74.29	75.99	73.43	74.69	0.82	9.44
	24	KNN	82.81	82.81	83.46	83.14	0.88	75.24	75.96	76.19	76.07	0.76	7.57
	25	LR	84.13	94.25	84.25	84.25	0.89	71.43	70.18	77.73	73.76	0.74	12.7
	26	SGDC	82.94	80.43	87.4	83.77	0.88	62.30	62.05	69.59	65.60	0.67	20.64
	Mean (μ _ML_)		83.40	84.44	86.22	83.98	0.89	70.82	71.05	74.24	72.53	0.75	12.58
	SD (σ _ML_)		±0.55	±5.75	±2.52	±0.60	±0.02	±5.11	±5.71	±3.09	±4.08	±0.05	±4.56

ACCU: accuracy; PREC: precision; REC: recall; FS: F1-score; AUC: area under the curve; SD: standard deviation; Xmer: transformer; Tr: train; Te: test; PD: percentage difference between the accuracy of seen and unseen.

**Table 3:** Benchmarking table highlighting attributes of scientific validation.

SN	Study and Citations	Feature Selection	NF*	Classifier Types	Augu*	Attn	UnSe*	Expl*	Gen*	Rel*	CV	GPU Clu*	uACCU* (%)	sACCU* (%)	uAUC* (0–1)	sAUC* (0–1)
1	Huang et al. [15]	DEA, TWAS	3	LR	✘	✘	✓	✘	✘	✘	K5	✘	66.00	69.23	0.71	0.79
2	Yifan et al. [13]	DEA	8	RF	✘	✘	✓	✘	✘	✘	K15	✘	61.70	75.00	0.73	0.85
3	Xie et al. [14]	DEA, SVM-RFE	3	SVM	✘	✘	✓	✘	✘	✘	K5	✘	87.41	89.28	0.83	0.96
4	Saeed et al. [11]	Manual	19	ANN	✘	✘	✘	✘	✘	✘	✘	✘	✘	89.73	✘	0.80
5	Fang et al. [16]	RFE	15	SVM	✘	✘	✓	✘	✘	✘	K5	✘	84.60	86.00	0.92	0.86
6	Peng et al. [30]	WGCNA, DEA, RFE	12	SVM	✘	✘	✓	✘	✘	✘	✘	✘	75.58	94.59	0.81	0.99

NF*: no of features; Augu*: augmentation; Attn: attention; UnSe*: unseen; Expl*: explainability; Gen*: generalization effect; Rel*: reliability test; CV: cross-validation; GPU Clu*: graphical processing unit cluster; uACCU*: unseen accuracy; uAUC*: unseen area-under-the-curve; DEA: differential expression analysis; TWAS: transcriptome-wide association study; SVM: support vector machine; RFE: recursive feature elimination; WGCNA: weighted gene co-expression network analysis; sACCU*: seen accuracy; sAUC*: seen area-under-the-curve.

## Data Availability

The gene expression datasets used in this study are publicly available from the NCBI Gene Expression Omnibus (GEO) repository. The accession number GSE57345 and GSE66360 contains transcriptomic profile related to cardiovascular conditions. This dataset was used for model training, validation, and performance evaluation. This dataset can be accessed at https://www.ncbi.nlm.nih.gov/geo/, accessed on 15 January 2026. The code used during the current study is not publicly available due to its proprietary nature, but the corresponding author can be reached for discussions.

## Supplementary Materials

The following supporting information can be downloaded at: https://www.mdpi.com/article/10.3390/diagnostics16071052/s1, Supplementary Figure S1. Architecture block diagram of Hybrid Deep Learning Models using xLSTM framework, Figure S2. Architecture of DEA, Figure S3. Sensitivity Analysis of wBio-GenAI to log_2_FC Thresholds, Figure S4. Go Biological Process Analysis, Figure S5. KEGG pathway Analysis.

## Appendix A. Performance Metrics

Appendix A summarizes the performance evaluation metrics used to assess the effectiveness of the proposed model. These metrics are derived from the confusion matrix components. Together, these metrics ensure a robust and multi-dimensional evaluation of the model’s performance.

(A1) $$w\mathrm{TPR}\;(\mathrm{Recall})=\frac{wTP}{wTP+wFN}$$

(A2) $$w\mathrm{FPR}=\frac{wFP}{wFP+wTN}$$

(A3) $$w\mathrm{TNR}\;(\mathrm{Specificity})=\frac{wTN}{wTN+wFP}$$

(A4) $$w\mathrm{FNR}=\frac{wFN}{wFN+wTP}$$

(A5) $$wA=\frac{wTP+wTN}{wTP+wTN+wFP+wFN}$$

(A6) $$wP=\frac{wTP}{wTP+wFP}$$

(A7) $$wF1-\mathrm{score}=\frac{2\times wP\times wR}{wP+wR}$$

(A8) $$wM=\frac{1}{N}\sum_{i=1}^{N}x_{i}$$

Here, x_i_ are the data points, N is the total number of data points; *w*TP are the true positive values; *w*FP are the false positive values; *w*TN are the true negative values; *w*FN are the false negative values; *w*TPR, *w*FPR, *w*TNR, *w*FNR, *w*𝒜, wP, wR, and *wM* represent true positive rate, false positive rate, true negative rate, false negative rate, accuracy, precision, recall, and mean (average), respectively.

## Appendix B. Acronym Table

**SN**	**Abbn***	**Description**	**SN**	**Abbn***	**Description**
1	ACCU	Accuracy	27	LASSO	Least Absolute Shrinkage and Selection Operator
2	ActFun	Activation Function	28	LSTM	Long Short-Term Memory
3	AI	Artificial Intelligence	29	LSTMu	LSTM Units
4	ANN	Artificial Neural Network	30	ML	Machine Learning
5	AUC	Area Under the ROC Curve	31	NS	Neighborhood Search
6	BCE	Binary Cross-Entropy	32	Opt	Optimizer
7	Bi	Bidirectional	33	PI	Percentage Improvement
8	BlkE	Block Effect	34	PREC	Precision
9	BS	Batch Size	35	REC	Recall
10	cLSTM	Conventional Long Short-Term Memory	36	RF	Random Forest
11	CNN	Convolutional Neural Network	37	RFB	Random Forest Boruta
12	CV	Cross-Validation	38	ROC	Receiver Operating Characteristic
13	DEA	Differential Expression Analysis	39	RFE	Recursive Feature Elimination
14	DEGs	Differentially Expressed Genes	40	SDL	Solo Deep Learning
15	DL	Deep Learning	41	SGDC	Stochastic Gradient Descent Classifier
16	DOut	Dropout Rate	42	SMOTE	Synthetic Minority Oversampling Technique
17	FC	Fold Change	43	SVM	Support Vector Machine
18	F1-Score FS	Harmonic Mean of Precision and Recall	44	TPR	True Positive Rate
19	FPR	False Positive Rate	45	TNR	True Negative Rate
20	GAN	Generative Adversarial Network	46	TWAS	Transcriptome-Wide Association Study
21	GenE	Generalization Effect	47	Uni	Unidirectional
22	GSE	Gene Expression Omnibus Series	48	Val	Validation Split
23	HDL	Hybrid Deep Learning	49	xLSTM	Extended Long Short-Term Memory
24	HF	Heart Failure	50	xLSTMcg	Cross-Gated Extended Long Short-Term Memory
25	KNN	K-Nearest Neighbors	51	xLSTMeg	Exponential-Gated Extended Long Short-Term Memory
26	LR	Logistic Regression	52	XGBoost	Extreme Gradient Boosting
Abbn*: Abbreviation.
