# Supplementary material for: Generative Artificial Intelligence vs. Transformer and Benchmarking Against Deep/Machine Learning: Classification and Scientific Validation of Heart Failure Patients Using Women’s Transcriptomic Gene Data
Source: Diagnostics (Basel). 2026 Apr 1;16(7):1052. doi: 10.3390/diagnostics16071052 (PMC13073481; doi:10.3390/diagnostics16071052)
Supplement: Supplementary file 1 [file diagnostics-16-01052-s001.zip › diagnostics-4201153-supplementary.pdf]

# Supplemental Materials (Not Included in Manuscript)

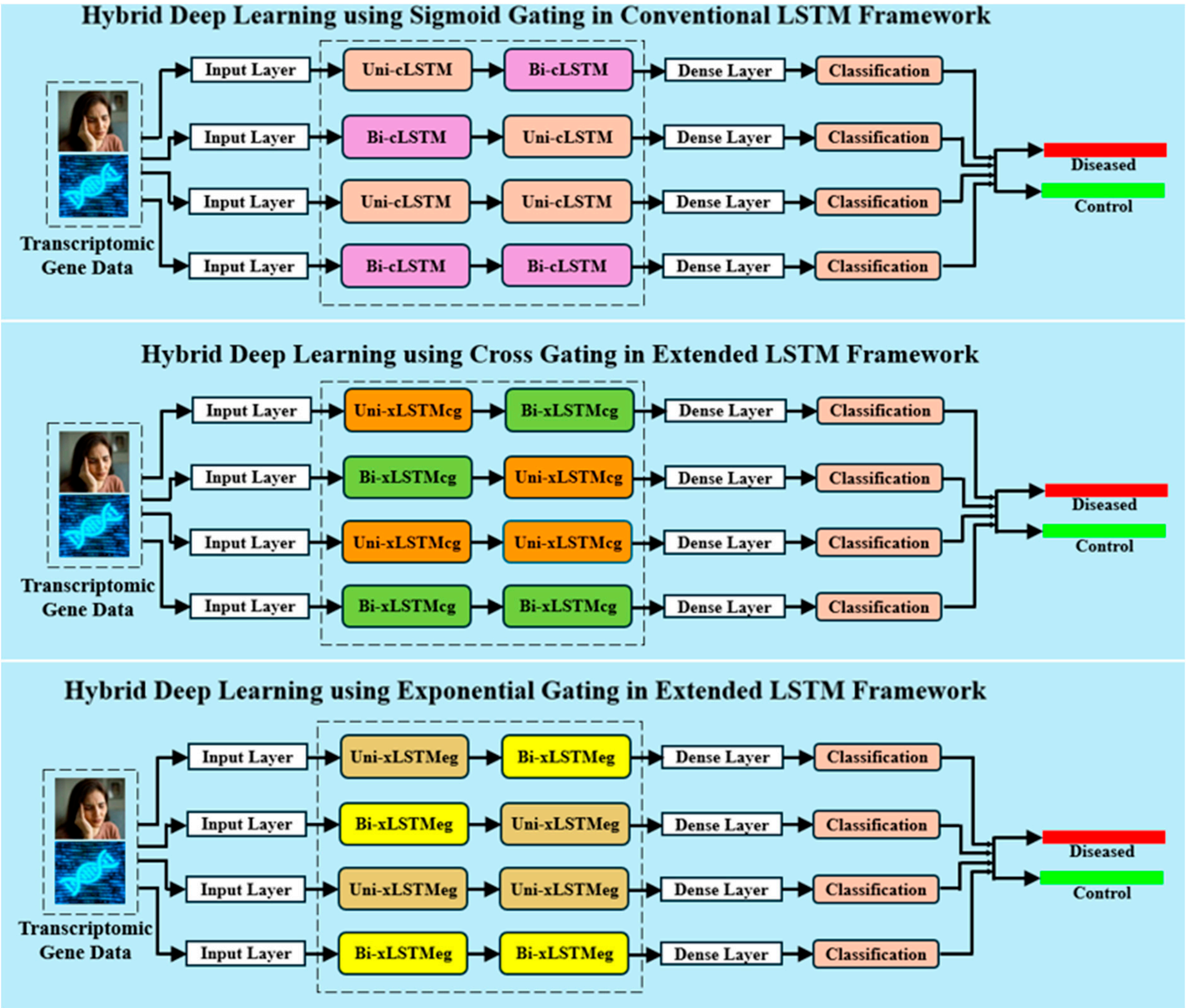


Fig. S.1. Architecture block diagram of Hybrid Deep Learning Models using xLSTM framework.

# Differential Expression Analysis

**1.1 Get the data: Take the gene expression data for all patients.**

- This step involves collecting raw gene data and performing initial preprocessing to remove genes with duplicate or null values.
- Normalization is applied to the raw data to ensure comparability across different patient samples before further analysis.

**1.2 Label the patients: Mark healthy as 0, diseased as 1.**

- Samples are categorized using binary encoding where "Control" subjects are labeled as 0 and "Diseased" subjects (such as those with HCM or AMI) are labeled as 1.
- This categorical labeling is essential for supervised feature extraction and subsequent training of the transformer models.

### 1.3 Separate data: Split into healthy group and diseased group.

- The pre-processed patient data is partitioned based on their assigned class labels into distinct groups for comparative statistical testing.
- This separation allows the algorithm to perform fold-wise analysis independently within a cross-validation framework to ensure no test data leakage.

### 1.4 Check each gene:

- Find average expression in both groups: The mean expression level for every individual gene is calculated separately for the healthy control group and the diseased group.
- Calculate fold change (how much it goes up or down): The Fold Change (FC) is computed by determining the ratio or difference between the mean expression in the disease group versus the control group.
- Run a t-test to see if the difference is real, not random: A t-test is performed for each gene to evaluate the statistical significance of the observed expression differences between the two groups.

### 1.5 Pick important genes: Keep only genes with big changes & strong statistical evidence.

- Genes are filtered based on strict thresholds, specifically requiring an adjusted *p-value* of less than 0.05 ($<0.05$) to ensure statistical significance.
- A magnitude threshold is also applied, where only genes with an absolute $Log_2FC >= 1.5$ are retained to capture biologically meaningful changes.

### 1.6 Classify: Mark them as Upregulated or Downregulated.

- Genes that pass the significance filters are classified as "Upregulated" if their expression is significantly higher in the diseased group ($Log_2FC >= 1.5$).

- Conversely, genes are marked as "Downregulated" if their expression is significantly lower in the diseased group compared to the control group.

### 1.7 Save results: Put the final list onto a table.

- The selected Differentially Expressed Genes (DEGs), along with their $log_2FC >= 1.5$ and adjusted *p-values*, are compiled into a final feature list.
- These results serve as the "High Risk Genes" which are then validated through biological pathway enrichment analyses like Gene Ontology (GO) and Kyoto Encyclopedia of Genes and Genomes (KEGG).

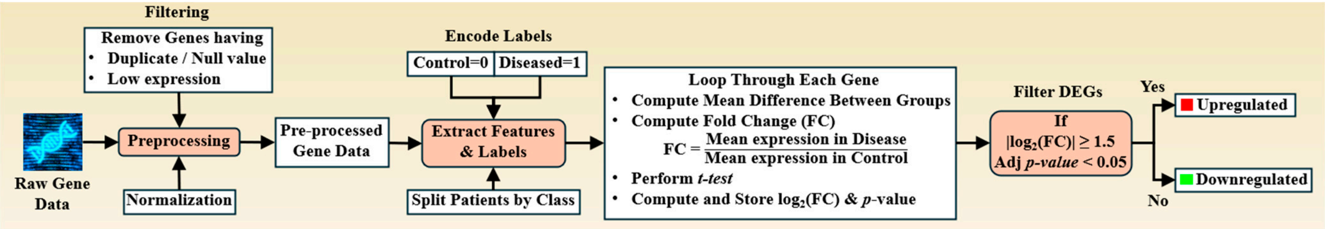


Fig. S 2. Architecture of DEA.

## Sensitivity of Model Performance to Varying log$_2$FC Thresholds

To assess robustness, we conducted a comprehensive sensitivity analysis using wBio-GenAI by varying the log$_2$FC threshold while keeping the adjusted p-value fixed at $< 0.05$. The evaluated thresholds include log$_2$FC $\geq$ 0.3, 0.5, 0.8, 1.2, 1.5, 1.6, 1.7, 1.8, and 2.0, representing progressively relaxed to stringent effect-size criteria.

Crucially, differential expression analysis (DEA) was performed independently within each training fold of the 10-fold cross-validation framework. For every threshold, the complete pipeline—DEA, feature encoding, class balancing, and model training—was executed using training data only, thereby ensuring that no test data influenced feature selection or model optimization.

The results reveal a monotonic increase in accuracy from lower thresholds up to log$_2$FC $\geq$ 1.5, where the highest accuracy (98.11%) is achieved. This trend reflects progressive removal of weakly informative genes and an improvement in signal-to-noise ratio. Beyond log$_2$FC $\geq$ 1.5, performance declines gradually, with a pronounced drop observed at log$_2$FC $\geq$ 2.0. This inverted-U shaped performance curve empirically demonstrates that the proposed framework is stable and robust across a wide range of DEG thresholds, rather than being dependent on a narrowly chosen cutoff.

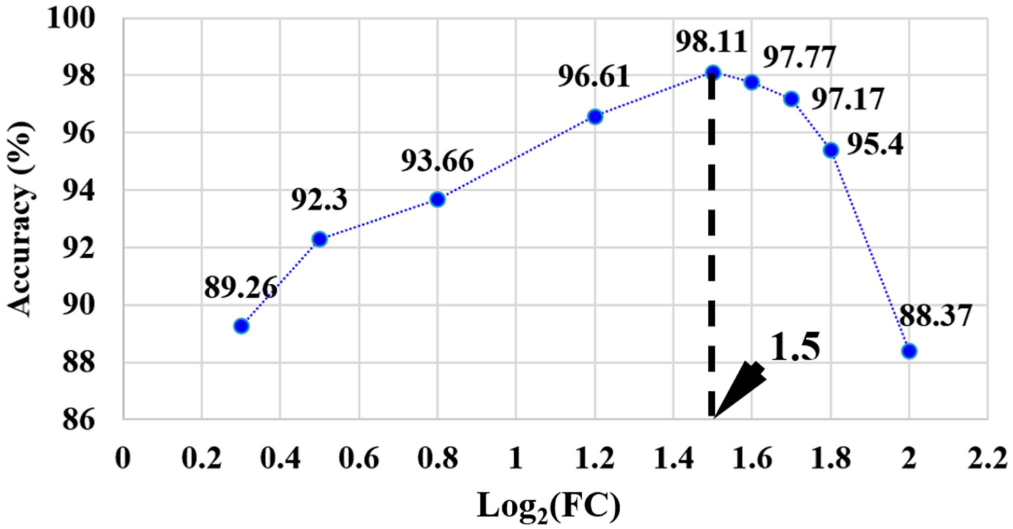


Fig. S 3. Sensitivity Analysis of wBio-GenAI to log$_2$FC Thresholds

**Algorithm: Sensitivity Analysis of a wBio-GenAI to log$_2$FC Thresholds**

*Input:*

Dataset D with gene expression matrix X and labels y
log$_2$FC thresholds T = {0.3, 0.5, 0.8, 1.2, 1.5, 1.6, 1.7, 1.8, 2.0}
Adjusted p-value cutoff $\alpha = 0.05$
Number of folds K = 10

*Output:*

**// Performance metrics for each log$_2$FC threshold**
For each threshold t in T do:
  Initialize performance list P_t ← empty
  Perform K-fold cross-validation on D:
    For each fold k = 1 to K do:
      Split D into training set D_train^k and test set D_test^k
      **// Fold-wise differential expression analysis**

Perform DEA on D_train^k
Select genes G_t^k such that:
adjusted p-value < α
$|\log_2 FC| \geq t$
**// Feature processing and model training**
Encode D_train^k using selected genes G_t^k
Apply class balancing on D_train^k
Train the model on processed D_train^k
**// Evaluation**
Encode D_test^k using G_t^k
Evaluate trained model on D_test^k
Store performance metric in P_t
Compute mean performance across folds for threshold t
Return performance metrics across all thresholds T

## High Risk Genes

The following genes are the common genes across HCM and AMI datasets. These genes were extracted using DEA.

"SERPINA3", "PLA2G2A", "ASPN", "FCN3", "SFRP4", "IL1RL1", "NPPA", "MYH6", "HBB", "CD163", "SERPINE1", "LYVE1", "FRZB", "EIF1AY", "OGN", "VSIG4", "CYP4B1", "COL14A1", "LUM", "MXRA5", "ADAMTS4", "SMOC2", "ADAMTS9", "AOX1", "RNASE2", "MGST1", "AREG", "HMGCS2", "IFI44L", "ANKRD2", "USP9Y", "METTL7B", "MYOT", "PHLDA1", "MNS1", "FREM1", "SFRP1", "FNDC1", "PI16", "PDE5A", "HAPLN1", "MME", "S100A8", "C6", "ECM2", "HBA2", "PTN", "TUBA3D", "C1QTNF1", "NRK", "DSC1", "ANPEP", "NAP1L3", "FCER1G", "SGPP2", "PI15", "FKBP5", "ALOX5AP", "RARRES1", "AQP3", "TLL2", "CNN1", "GUCA1C", "CCL2", "SPP1", "PTX3", "IL1R2", "DDX3Y", "LCN6", "PLEKHH2", "UTY", "ASB14", "FMOD", "TTTY10", "CRISPLD1", "CRYM", "STAT4", "SCN2B", "SHISA3", "OMD", "MATN2", "OLFML1", "MFAP4", "NEB", "TMEM71", "GFPT2", "LAPTM5", "SLC16A9", "RGS4", "CHRDL1", "MT1A", "LRRC17", "TUBA3E"

***Global Pathway-Level Validation Using GO and KEGG Analyses***
To move beyond local explanations, we performed global pathway enrichment analyses using FDR-adjusted p-values.
*GO Biological Process Enrichment*
Significantly enriched GO terms include:

- Extracellular matrix organization (GO:0030198)
- Extracellular structure organization (GO:0043062)
- Neutrophil activation and degranulation (GO:0002283, GO:0043312)
- Complement activation, lectin pathway (GO:0001867)
- Regulation of vascular wound healing (GO:0061043)
- Replicative senescence (GO:0090399)

These processes directly correspond to genes identified by LIME (e.g., SERPINA3, S100A8, FCER1G, SMOC2, OMD), demonstrating that LIME-identified features converge on coherent immune-inflammatory and vascular remodeling pathways.

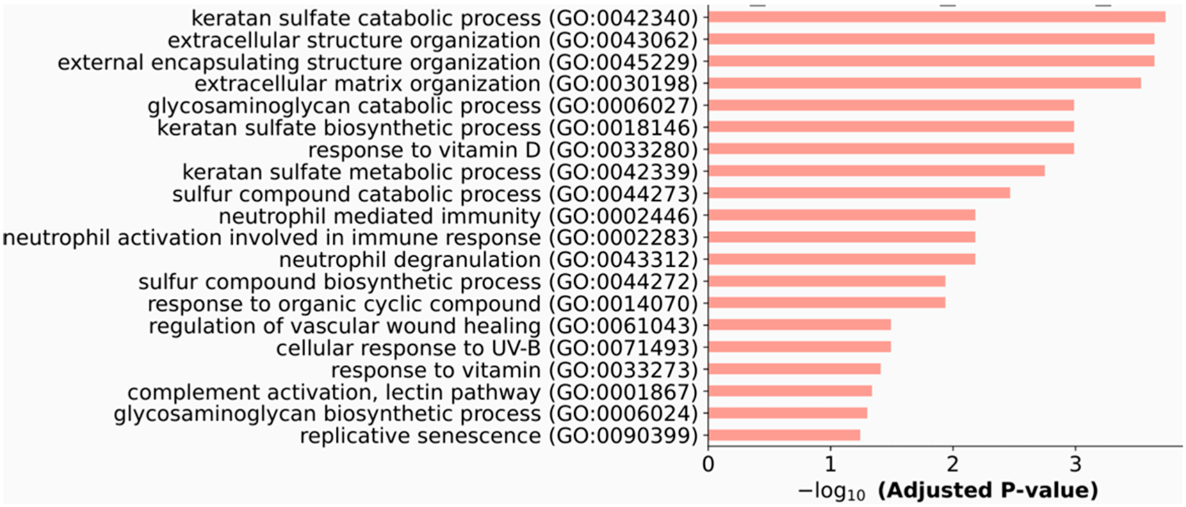


Fig. S 4. Go Biological Process Analysis.

*KEGG Pathway Enrichment*

KEGG analysis further supports this convergence, identifying enrichment in:

- Complement and coagulation cascades
- Renin–angiotensin system
- Fluid shear stress and atherosclerosis
- Glutathione metabolism and oxidative stress pathways
- Hematopoietic cell lineage

These pathways are well-established in cardiovascular and inflammatory disease progression and provide mechanistic grounding for the genes highlighted by the model.

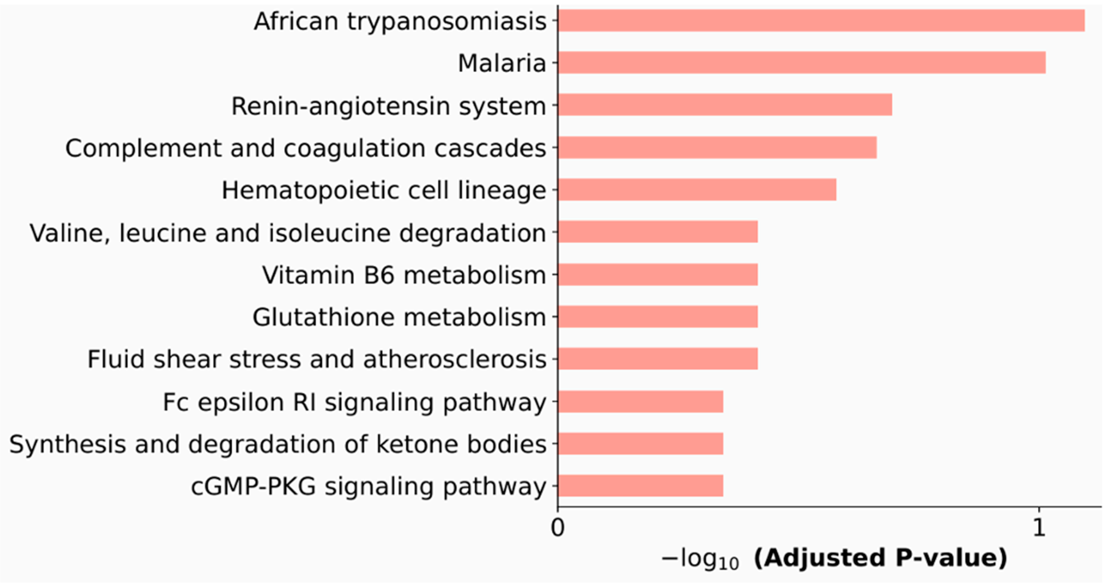


Fig. S 5 KEGG pathway Analysis.
